# A Locally Executable AI System for Improving Preoperative Patient Communication: Multidomain Clinical Evaluation

**DOI:** 10.2196/89173

**Published:** 2026-07-21

**Authors:** Motoki Sato, Sou Nagata, Mizuho Ohnuma, Hidekazu Takahashi, Tomoaki Kakazu, Masayuki Yamamura, Atsushi Yoshikawa, Yuki Matsushita

**Affiliations:** 1 Department of Skeletal Development and Regenerative Biology, Graduate School of Biomedical Sciences, Nagasaki University, 3F Building for Basic Dental Science, 1-7-1 Sakamoto, Nagasaki, Japan, 81 95-819-7633, 81 95-819-7633; 2 Boston Medical Sciences, Inc., Tokyo, Japan; 3 Digestive Diseases Center, Showa University Koto Toyosu Hospital, Tokyo, Japan; 4 Department of Computer Science, School of Computing, Institute of Science Tokyo, Tokyo, Japan; 5 College of Informatics, Kanto Gakuen University, Yokohama, Kanagawa, Japan

**Keywords:** AI, artificial intelligence, large language models, natural language processing, privacy, energy metabolism, doctor-patient communication, implementation science

## Abstract

**Background:**

Patients undergoing invasive procedures frequently experience anxiety and often have unanswered questions regarding the procedure. Although large language models show considerable promise for supporting patient communication in many cases, their deployment in health care is limited by the risk of hallucinations, data-privacy constraints, and high energy costs—factors that impede equitable access in resource-limited settings.

**Objective:**

This study aims to develop and evaluate LENOHA (Low Energy, No Hallucination, Leave No One Behind Architecture), a locally executable dialog system for safe, equitable, and sustainable preprocedural communication.

**Methods:**

We built expert-curated FAQ (frequently asked question) databases and independent test sets for 2 domains (tooth extraction and gastroscopy; 200 utterances per domain: 100 clinical questions and 100 casual). A sentence-transformer classifier routed inputs: clinical questions were answered verbatim from the vetted FAQs (nongenerative path), while casual conversation was handled by a locally hosted 8-billion-parameter small language model (Swallow-8B). We evaluated 4 sentence-transformer models (including E5-large-instruct) against cloud large language models (ChatGPT [GPT-4o] and Gemini Advanced) using accuracy, *F*_1_-score, and area under the receiver operating characteristic curve, and measured the on-device inference energy on a consumer graphics processing unit (RTX 3080).

**Results:**

Across both domains (N=400), E5-large-instruct achieved an accuracy of 98.3% (393/400; 95% CI 96.4%‐99.1%) and an area under the curve of 0.996, with only 7 out of 400 (1.8%) misclassifications. This performance was not statistically different from that of ChatGPT (GPT-4o), which had 6 out of 400 (1.5%) errors (McNemar test with Holm adjustment; *P*>.99). Sustainability measurements showed approximately 2.23 mWh per request (latency≈0.10 s; video RAM≈2.2 GiB average, ≈2.5 GiB peak) for the nongenerative clinical path vs approximately 168.27 mWh (latency≈8.51 s; video RAM≈13.3 GiB average, ≈14.0 GiB peak) for small language model small talk—approximately a 75-fold higher energy footprint per reply for the generative path.

**Conclusions:**

High-precision, nongenerative clinical support is feasible using local, low-cost hardware without cloud dependence. By decoupling clinical information retrieval from generative chitchat, LENOHA enhances safety, preserves privacy, and markedly reduces energy use, offering a practical blueprint for sustainable and equitable medical AI deployment across diverse care settings.

## Introduction

Patients undergoing invasive medical procedures, such as tooth extraction or upper gastrointestinal endoscopy (gastroscopy), commonly experience preprocedural questions and anxiety [1,2]. Providing patients with appropriate information and reassurance through effective communication is critical for building trust and promoting active engagement in treatment, which are essential factors in successful clinical care [1,2]. Interventions, such as educational materials and structured communication strategies, have been shown to significantly enhance patient understanding, satisfaction, and involvement in decision-making [3].

Digital technologies have been shown to improve early comprehension of surgical informed consent without increasing anxiety or reducing satisfaction [4].

In today’s high-pressure clinical environments, it is not always feasible for health care professionals to provide fully personalized explanations to every patient. In Japan, physician shortages in rural areas and surgical specialties limit the time available for thorough preprocedural communication, while patients often hesitate to ask questions for fear of burdening already busy staff [5]. Prior studies have shown that presenting multimedia or electronic consent materials before the consultation can reduce face-to-face explanation time by approximately 30% to 60% while maintaining patient understanding, anxiety levels, and satisfaction, suggesting that such approaches can improve time efficiency for both patients and health care professionals [6]. Moreover, microcosting analyses from the UK National Health Service indicate that digital consent pathways can be at least cost-neutral and, in some scenarios, slightly less expensive compared with paper-based workflows [7].

In recent years, large language models (LLMs) have demonstrated remarkable potential in patient education, psychological support, and workflow optimization [8]. For example, recent evidence suggests that AI-based LLMs offer a promising avenue for improving the quality and readability of oral surgery informed consent documents, outperforming conventional web-based materials in standardized assessments [9]. However, the deployment of LLMs in real-world health care contexts poses several challenges.

First, ensuring the reliability and safety of the information generated by LLMs is a paramount concern [10]. LLMs are inherently prone to generating factually incorrect content, referred to as “hallucinations,” and may reproduce biases present in their training data, potentially exacerbating existing health disparities and posing significant risks to patient safety [10,11].

From a theoretical perspective, recent work has shown that hallucinations cannot be eliminated for any computable LLM, even with more parameters, more data, or sophisticated prompting, indicating that some residual risk of incorrect output is inherent to the technology [12].

Retrieval-augmented generation (RAG) has emerged as a promising strategy to enhance reliability by referencing external knowledge bases [13]. RAG itself is not a panacea and has inherent challenges, such as the “lost-in-the-middle” problem, where LLMs may fail to use retrieved information effectively [14]. Consequently, recent research has shifted toward architectural safeguards that constrain how LLMs access and use external information. One representative example is the Model Context Protocol (MCP; Anthropic), which standardizes how language models ingest and isolate external data, thereby decoupling stochastic reasoning from deterministic execution [15,16]. For instance, a recent study by Avila et al [17] demonstrated that using an MCP-based architecture in structural analysis reduced predictive deviations from over 400% (in unconstrained LLMs) to under 1.5% [17]. Furthermore, such architectural compartmentalization ensures reproducibility and traceability, which are critical requirements for high-stakes environments. These findings strongly indicate that rigid architectural constraints, rather than basic RAG or prompt engineering, are essential for maintaining rigorous safety standards.

Second, practical barriers to deployment hinder equitable access to AI. The reliance on cloud-based APIs for most leading general-purpose LLMs raises substantial privacy and security concerns, whereas their high computational and energy demands render local execution infeasible for many health care facilities, contributing to a significant environmental footprint. Health care systems are already responsible for 4% to 5% of global greenhouse gas emissions, making the environmental impact of AI a pressing issue [18,19]. As highlighted in a recent review by Ueda et al [20], the rapid expansion of AI in Japanese health care necessitates a shift toward sustainable practices to mitigate its environmental impact, including greenhouse gas emissions from intensive computing resources [20]. Furthermore, avenues for accessibility face their own equity challenges; automatic speech recognition (ASR) systems exhibit performance disparities across diverse accents [21,22], and recent studies show that leading LLMs, such as ChatGPT (GPT-4o), produce clinical vignettes that stereotype demographic presentations and alter recommendations based on patient race and gender [23]. These findings underscore the critical need for novel architectural approaches that explicitly prioritize safety, equity, and environmental sustainability. While large models dominate the current AI discourse, Jeanquartier et al [24] emphasized that small language models (SLMs) offer significant promise for application scenarios where resource efficiency and data privacy are paramount, such as in offline medical devices. By circumventing the massive computational costs of larger models, SLMs provide a more accessible and sustainable pathway for health care informatics. Rather than pursuing incremental performance gains within the existing flawed holistic evaluation framework, this paper proposes an architectural framework—a blueprint designed from first principles to be safe, equitable, and sustainable through the integration of “eco-design” and local execution.

Third, a holistic framework is required for the ethical and sustainable implementation of AI in health care. Morley et al [19] recently advocated for a systems approach, proposing five core infrastructural requirements for ethical AI implementation: (1) robust data exchange, (2) epistemic certainty with staff autonomy, (3) actively protected health care values, (4) validated outcomes with meaningful accountability, and (5) environmental sustainability. Similarly, to systematize ethical considerations, Ning et al [25] advocated for a comprehensive assessment checklist for generative AI research grounded in 9 core principles: accountability, autonomy, equity, nonmaleficence, privacy, security, integrity (in medical education and quality of clinical research), transparency, and trust [25]. These multifaceted challenges underscore the need for new architectural approaches that prioritize safety, equity, and practical applicability. Building upon these foundations to address the specific needs of resource-constrained settings, the recently developed SAFE-AI (Scalable Agile Framework for Execution in AI) framework by Nemteanu et al [26] emphasizes the necessity of embedding lightweight, “minimum necessary safeguards” and risk interpretability directly into the AI development life cycle.

To overcome these limitations, this study adopts a different approach. We introduce the LENOHA system (Low Energy, No Hallucination, Leave No One Behind Architecture). Its underlying principle aligns with the fundamentals of sustainable AI, particularly in settings constrained by limited infrastructure and resources. To evaluate the utility and generalizability of this architecture, we constructed independent test datasets from 2 distinct clinical domains—oral and maxillofacial surgery (tooth extraction) and gastroenterology (gastroscopy)—and conducted a comparative performance assessment across multiple model architectures, including open-source sentence-transformer (ST) models and commercial LLMs. The results demonstrate that high-precision classification is achievable even on modest local hardware, offering a feasible and scalable solution to one of the most pressing bottlenecks of health care AI.

The primary aim of this study was to develop and evaluate LENOHA, a locally executable dual-pathway architecture that routes clinical queries to a nongenerative, clinician-curated FAQs (frequently asked questions) pathway and casual conversations to a local generative model.

We assessed whether this design can reliably separate clinical from casual utterances in preprocedural settings while reducing privacy risks and operational energy compared to a fully generative approach.

## Methods

### Study Design and Clinical Domains

In this study, we focused on a single clinical problem: preprocedural patient communication for invasive but relatively low-risk procedures. We explicitly followed a stepwise AI life cycle approach, focusing on the early development and technical validation phases rather than the in-workflow clinical deployment. Consistent with stepwise implementation models, such as those of van de Sande et al [27] and broader life cycle concepts proposed by Kuziemsky et al [28], this study corresponds to phase 1 (AI model development) and phase 2 (assessment of AI performance and reliability) and does not yet encompass phase 3 clinical testing with actual patients. To test whether our architectural approach can be generalized across heterogeneous clinical settings, we deliberately selected 2 distinct domains—oral and maxillofacial surgery (tooth extraction) and gastroenterology (gastroscopy)—that differ in specialty, workflow, and risk framing, while sharing the same need for scalable preprocedural counseling. For each domain, we prepared 3 resources:

- An expert-curated FAQ database was used as the target of the ST-based matcher.
- A validation dataset was used to determine the operating thresholds.
- An independent test dataset was used for the final evaluation.

Utterances classified as clinical questions were routed to the nongenerative FAQ path, and utterances classified as casual conversation were intended to be handled by a locally executed SLM.

**Figure 1.**
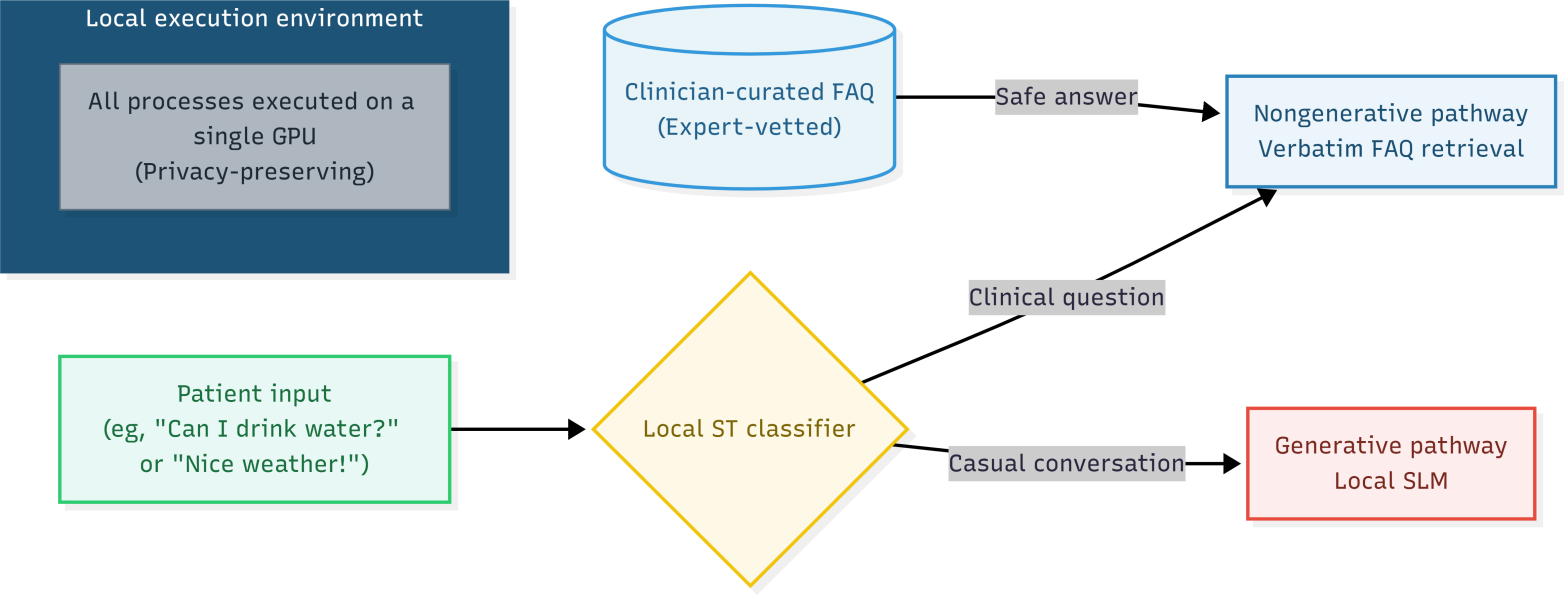
Schematic overview of the LENOHA system data flow. All processing was performed locally on a single graphics processing unit (GPU) to preserve patient privacy. A local sentence-transformer (ST) classifier first categorized the patient input. Inputs identified as clinical questions were routed to a nongenerative pathway, which retrieved a verbatim, clinician-curated answer from the frequently asked question (FAQ) database, minimizing the risk of hallucination. In contrast, casual conversations were routed to a generative pathway, where a local small language model (SLM; Swallow-8B) produced an empathetic response.

A schematic overview of the system’s data flow is shown in Figure 1.

### FAQ Databases

#### Tooth Extraction

Under the supervision of dentists and oral and maxillofacial surgeons, we defined categories based on FAQs observed in routine Japanese practice (eg, travel, postoperative daily life, jaw or mouth opening, anxiety, anesthesia, dysesthesia, return to work, bleeding, diet, swelling, pain, indication, procedure time, bone resection, medication, temporomandibular joint disorder, infection, cost, postoperative prosthetics, pregnancy or breastfeeding, comorbidities, cancellation or interruption, presence of wisdom teeth, tooth longevity, and suture removal). The complete clinical schema template used for this categorization is provided in Multimedia Appendix 1.

For each category, we collected multiple colloquial paraphrases in standard Japanese rather than a single template to reflect real patient utterances. In total, we compiled approximately 4000 domain-specific questions, which substantially exceeded the coverage reported in previous FAQ-style systems and was intended to reduce false negatives during inference.

#### Gastroscopy

Using the tooth extraction schema (Multimedia Appendix 1) as a template and in consultation with physicians and endoscopists, we adapted the categories to the endoscopy setting: items that were not relevant (eg, “jaw”) were removed, and endoscopy-specific topics (eg, *Helicobacter pylori*, preparation, sedation or throat anesthesia, infection control, postexamination driving or work, emergency contact, and rescheduling) were added. The final endoscopy FAQ contained approximately 1600 clinician-curated questions, each paraphrased in standard Japanese.

### Definitions

To rigorously separate utterances that require medical oversight from those that do not, we defined 2 operational categories that were applied across both domains. This distinction was designed to filter out LLM hallucination risks for clinical content while still enabling supportive replies for nonclinical talk.

#### Clinical Question

Utterances explicitly seeking medical, clinical, or practical judgments and information require a clinically consistent and institutionally aligned response. These utterances were considered unsafe for free-form LLM generation.

Examples of intent included questions about postprocedural life, work, or diet; medical concerns regarding anesthesia, bleeding, pain, or infection; specific logistical queries (such as duration, cost, and precautions); consultations regarding regular medications, comorbidities, pregnancy, or breastfeeding; and requests for specific medical advice from health care staff.

#### Casual Conversation (Small Talk)

Utterances without clinical intent that are not mapped to any FAQ category.

Examples of intent include greetings, weather discussions, general small talk, personal updates (excluding medical symptom consultations), light expressions of gratitude, and simple expressions of emotion (eg, “I’m nervous” and “I’m scared”) that do not explicitly ask for medical coping strategies.

### Operational Rule and Decision Boundary

The critical criterion for classification was whether the speaker expected a clinically aligned medical response. For instance, the simple emotional expression, “I am nervous” is classified as Casual (Small Talk), whereas “Are there any ways to ease my nerves?” is classified as a clinical question requiring a safe, vetted response. The classifier’s operational rule was strictly defined: if an utterance did not match any clinical or FAQ category above the confidence threshold, it was treated as casual conversation.

### Synthetic Validation and Test Datasets

#### Rationale

Because the purpose of this study was to benchmark the core architecture (classifier+routing) and not to evaluate the deployment quality in a single hospital, we used expert-supervised synthetic text rather than deidentified patient records. This approach avoided privacy concerns and removed confounders related to the recording style.

Crucially, based on our preliminary internal validation, we observed that the semantic decision boundary between “medical queries” and “casual conversation” is highly sensitive to linguistic nuances. To rigorously evaluate the architectural feasibility of our novel routing logic without confounding variables, we established a study protocol to validate the system using standardized Japanese before introducing linguistic variations, such as dialects. This approach ensured that the baseline performance reflected architectural capability rather than linguistic noise.

#### Validation Datasets

For each domain, we first generated candidate utterances using multiple frontier LLMs (Groq Compound-Beta; Llama-3.1-Nemotron-Ultra-253B-v1, NVIDIA Corporation; Google Gemma-3-27B-IT; Mixtral-8×7B, Mistral AI) to obtain a broad spectrum of Japanese sentences. To ensure transparency and reproducibility, the specific prompts used for data generation, including instructions to simulate diverse patient personas (eg, varying anxiety levels), are provided in Multimedia Appendix 2.

Two independent domain experts per field (dentists or oral surgeons and physicians or endoscopists), who were not involved in the prompt engineering process, reviewed, corrected, and labeled each utterance as either a clinical question or a casual conversation. This process yielded 400 validation utterances per domain (200 clinical and 200 casual). These validation sets were used only to set the operating thresholds for the ST classifiers.

#### Test Datasets

To ensure independence from the construction and threshold-tuning phases, we generated test candidates using different LLMs (Qwen3-35B-A2 and Claude [Sonnet 4]), followed by the same rigorous review and labeling processes conducted by independent specialists. The final test datasets consisted of 200 utterances per domain (100 clinical and 100 casual). These test sets were the only data used for the final between-model comparisons.

To establish the reliability of the ground-truth labels, interreviewer agreement for the binary classification task (clinical vs casual) was evaluated, demonstrating perfect consensus (Cohen κ=1.0).

### Ethical Considerations

The study protocol was approved by the Ethics Committee of Nagasaki University (approval: 23090101 and 24092701). As all datasets consisted of synthetic utterances generated under expert supervision and contained no personal data from real patients, informed consent was not required and no identifiable information was collected or stored. The study design adhered to ethical guidelines for medical research involving human participants.

### Models

#### ST-Based Classifiers (Primary)

We evaluated 4 ST encoders: a Japanese-specific SBERT (sonoisa/sentence-bert-base-ja-mean-tokens), a lightweight multilingual MiniLM (paraphrase-multilingual-MiniLM-L12-v2), a high-performance multilingual E5-large (intfloat/multilingual-e5-large), and its instruction-tuned variant, E5-large-instruct. All FAQ items in each domain were embedded once more. Incoming patient utterances were then embedded using the same model, and the cosine similarities were computed. The maximum similarity score for each utterance was compared with a domain-specific threshold, which was objectively determined in the validation set using the Youden index.

#### LLM Baselines (Comparators)

For comparative context, we evaluated 2 representative frontier LLM services available between April 2025 and May 2025: ChatGPT (GPT-4o; OpenAI) and Gemini Advanced 2.5 Pro (Google). Both models were accessed through their web interfaces. They were provided with an identical prompt, which explicitly detailed the class definitions and required output formats, and were forced to output a single deterministic label (either clinical or casual). It is important to note that these baseline models were strictly evaluated as classifiers and were not used to generate synthetic test datasets.

#### Local SLM Path

A locally hosted Japanese SLM (Llama-3.1-Swallow-8B-Instruct) was used to test the feasibility of on-device small talk generation for utterances classified as casual. The decoding parameters were constrained to avoid clinical advice.

### Evaluation

#### Metrics

We calculated standard binary metrics: accuracy, precision, recall, *F*_1_-score, specificity, balanced accuracy, and receiver operating characteristic–AUC (area under the receiver operating characteristic curve; AUC only for ST models, as they output continuous scores; LLMs were treated as deterministic labelers).

#### Sample Size and Statistics

Each domain had a total of 200 test utterances (100 clinical and 100 casual). For an expected accuracy of approximately 0.95, the 95% Wilson CI half-width was approximately 0.03. Prespecified pairwise comparisons (E5-large-instruct vs the other STs vs both LLMs) were tested using 2-sided McNemar tests; the Holm adjustment was applied for multiplicity. No missing data were found.

## Results

### Overall Performance

The primary outcome of this study demonstrates that a locally executable ST classifier can approach the classification quality of leading cloud-based LLMs for the safety-critical task of routing patient utterances to the appropriate department. Furthermore, end-to-end local execution proved to be both highly safe and sustainable: no clinically unsafe content was generated under the constrained decoding settings for casual conversations, and the nongenerative clinical pathway (FAQ retrieval) was approximately 75 times more energy-efficient than the generative small-talk pathway.

### Validation Performance

#### Tooth Extraction

In the tooth extraction validation set (N=400; 200 clinical, 200 casual), all ST classifiers achieved high discrimination when the thresholds were optimized using the Youden index. E5-large (AUC=0.990; *F*_1_-score=0.965) and E5-large-instruct (AUC=0.989; *F*_1_-score=0.971) showed the best balance between sensitivity and specificity. SBERT also performed well (AUC=0.961; *F*_1_-score=0.930). Despite its small size, MiniLM remained usable (AUC=0.973) but showed a lower *F*_1_-score (0.825), indicating more false negatives in this domain. These validation thresholds were frozen and reused for test evaluation.

#### Gastroscopy

In the gastroscopy validation set (N=400), the performance was similarly strong.

E5-large-instruct achieved near-perfect classification (accuracy=0.988; recall=0.995; *F*_1_-score=0.988). E5-large followed closely (accuracy=0.965; *F*_1_-score=0.965). Lighter models such as SBERT (accuracy=0.928; *F*_1_-score=0.928) and MiniLM (accuracy=0.925; *F*_1_-score=0.926) still achieved acceptable performance, confirming that the classification task can be solved reliably across 2 clinically distinct domains. These thresholds were also applied in the test phase.

### Test Performance on Independent Data

Among the ST models, E5-large-instruct achieved the best overall performance across both clinical domains, with a mean accuracy of 0.983, a recall of 0.985, and an *F*_1_-score of 0.983, corresponding to 7 misclassifications out of 400 test utterances (Table 1). Discrimination remained high under distribution shift, with AUCs of 0.994 (tooth extraction) and 0.998 (gastroscopy), indicating a robust separation between clinical and casual inputs. The E5-large model also generalized well, showing only a marginal decrease relative to the instruction-tuned variant. In contrast, SBERT exhibited the largest degradation, particularly in the gastroscopy domain (accuracy=0.835), and produced the highest number of errors (51/400; Table 1). These results suggest that relying solely on a Japanese-specific ST model may be less robust when inputs reflect LLM-influenced phrasing or clinically structured language.

**Table 1.** Test performance metrics for the tooth extraction and gastroscopy domains, along with overall performance.

Model and domain	Accuracy	Recall	AUC^a^	Misclassification (FP^b^+FN^c^)^d^
SBERT^e^				
Tooth extraction	0.910	0.950	0.962	18 (13+5)
Gastroscopy	0.835	0.910	0.936	33 (24+9)
Overall^f^	0.873	0.930	0.949	51 (37+14)
MiniLM				
Tooth extraction	0.915	0.900	0.972	17 (7+10)
Gastroscopy	0.965	0.950	0.984	7 (2+5)
Overall^f^	0.940	0.925	0.978	24 (9+15)
E5				
Tooth extraction	0.930	0.960	0.991	14 (10+4)
Gastroscopy	0.965	0.940	0.994	7 (1+6)
Overall^f^	0.948	0.950	0.993	21 (11+10)
E5-large-instruct				
Tooth extraction	0.980	0.990	0.994	4 (3+1)
Gastroscopy	0.985	0.980	0.998	3 (1+2)
Overall^f^	0.983	0.985	0.996	7 (4+3)
Gemini				
Tooth extraction	1.000	1.000	N/A^g^	0
Gastroscopy	1.000	1.000	N/A	0
Overall^f^	1.000	1.000	N/A	0
ChatGPT (GPT-4o)				
Tooth extraction	1.000	1.000	N/A	0
Gastroscopy	0.970	0.9434	N/A	6 (6+0)
Overall^f^	0.985	0.9717	N/A	6 (6+0)

a AUC: area under the curve.

b FP: false positive.

c FN: false negative.

d The number of misclassifications represents the total number of FPs and FNs across both domains.

e SBERT: sonoisa/sentence-bert-base-ja-mean-tokens.

f Overall metrics for the sentence transformer models were calculated as the unweighted average of the domain-specific values.

g N/A: not applicable. AUC was not computed for large language model (LLM) baselines because, as stated in the Metrics section, LLMs were treated as deterministic labelers outputting discrete labels rather than continuous scores, making AUC not applicable.

**Figure 2.**
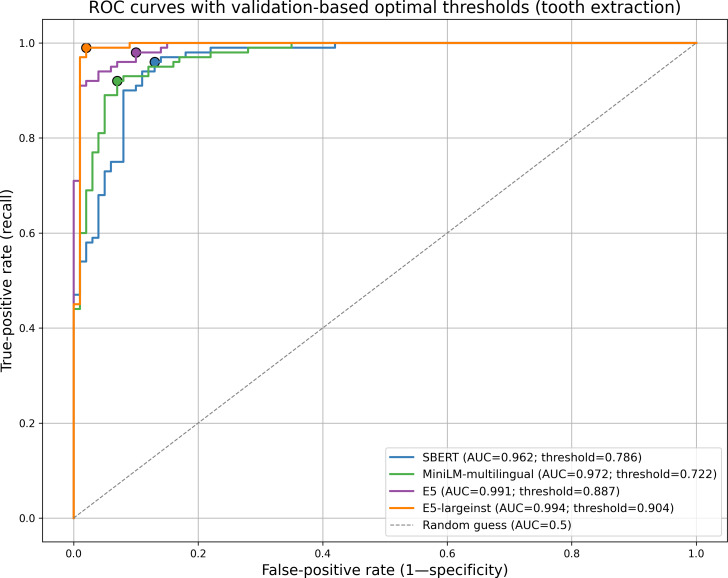
Receiver operating characteristic (ROC) curves with validation-based optimal thresholds (tooth extraction domain). AUC: area under the curve; SBERT: sonoisa/sentence-bert-base-ja-mean-tokens.

**Figure 3.**
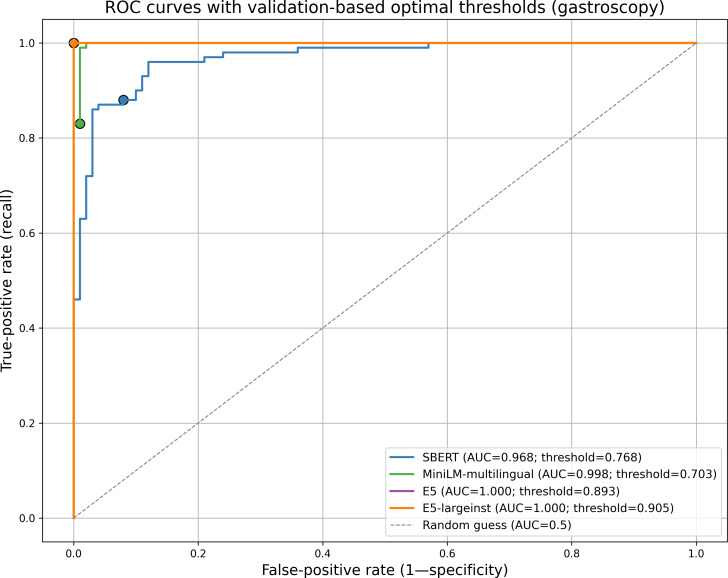
Receiver operating characteristic (ROC) curves with validation-based optimal thresholds (gastroscopy domain). AUC: area under the curve; SBERT: sonoisa/sentence-bert-base-ja-mean-tokens.

For frontier cloud LLMs, Gemini classified all the test items correctly (0 errors; Table 1). ChatGPT (GPT-4o) produced 6 errors, all of which were casual utterances misclassified as clinical (Table 1). Detailed performance metrics, including precision, specificity, and 95% CIs for all models, are provided in Multimedia Appendix 3. Notably, the error counts of ChatGPT (GPT-4o) (6/400) and the locally executable E5-large-instruct (7/400) were comparable. The receiver operating characteristic curves and validation-optimized operating thresholds are shown for the tooth extraction (Figure 2) and gastroscopy (Figure 3) domains.

### Statistical Comparison of Models

To rigorously assess the performance differences between the key models, we conducted pairwise McNemar tests with continuity correction (Table 2).

**Table 2.** Pairwise McNemar test results with effect sizes and statistical power.

Model 1	Model 2	b (model 1 only correct)	c (model 2 only correct)	*P* value	Cohen *h*	Power
SBERT^a^	MiniLM	18	45	<.001	0.8858	0.999
SBERT	E5	12	42	<.001	1.1781	1
SBERT	E5-large-instruct	4	48	<.001	2.0175	1
SBERT	ChatGPT (GPT-4o)	4	49	<.001	2.0284	1
SBERT	Gemini	0	51	<.001	3.1416	1
MiniLM	E5	13	16	.71	0.2073	0.124
MiniLM	E5-large-instruct	4	21	<.001	1.4955	1
MiniLM	ChatGPT (GPT-4o)	6	24	.002	1.287	0.999
MiniLM	Gemini	0	24	<.001	3.1416	1
E5-large	E5-large-instruct	1	15	.001	2.1309	1
E5-large	ChatGPT (GPT-4o)	6	21	.007	1.1781	0.991
E5-large	Gemini	0	21	<.001	3.1416	1
E5-large-instruct	ChatGPT (GPT-4o)	6	7	>.99	0.154	0.068
E5-large-instruct	Gemini	0	7	.02	3.1416	1
ChatGPT (GPT-4o)	Gemini	0	6	.04	3.1416	1

a SBERT: sonoisa/sentence-bert-base-ja-mean-tokens.

Compared with other ST models, E5-large-instruct demonstrated statistically significant superiority over SBERT (*P*<.001) and MiniLM (*P*<.001) with high statistical power (>0.999). Overall, these statistical results indicate that the local E5-large-instruct model achieves a classification performance comparable to that of the evaluated frontier cloud-based LLMs for this specific routing task.

Table 2 presents the results of pairwise comparisons between models using the McNemar test with continuity correction. For each model pair, the number of discordant cases, where only 1 model made a correct prediction, is reported as b (model 1 only correct) and c (model 2 only correct). The associated *P*-value indicates whether the difference in performance is statistically significant. Cohen *h* provides the effect size, and the post hoc power (assuming *α*=.05) reflects the likelihood of detecting such an effect with the observed sample size. Tests with low power (eg, power <0.8) may fail to detect true differences even if they exist (eg, comparisons involving MiniLM vs E5 and E5-large-instruct vs ChatGPT [GPT-4o]). In contrast, model pairs involving SBERT or Gemini consistently showed large effect sizes (Cohen *h*>0.8) and sufficient power (power≈1) indicate robust and meaningful performance differences.

### Local Small-Talk Generation

To check whether the nonclinical branch can also be kept on the device, we generated responses to 200 casual utterances with a local 8B Japanese SLM (Llama-3.1-Swallow-8B-Instruct-v0.3). All prompts produced coherent, context-appropriate replies, and no clinically unsafe content was observed under constrained decoding settings. The representative outputs are presented in Table 3.

Taken together with the classification results, this confirms that both clinical query filtering and nonclinical response generation can be run end-to-end on a single local graphics processing unit (GPU).

**Table 3.** Examples of casual conversation responses generated by Swallow-8B.

Patient’s casual utterance	Swallow-8B’s response
There is a lovely shop nearby.	Oh really? What type of shop is it?
A colleague at work has just gotten married.	Congratulations! That’s wonderful. Best wishes to them!
There is a great bakery in my neighborhood.	That sounds great! I have been looking for a new bakery. Do you have any recommendations for this?
The weather was pleasant that day.	Yes, the weather is beautiful today. A perfect day to go out. Do you have any plans?
I recently watched a funny variety show on TV.	I have been watching it too! That comedian’s act really made me laugh. Do you have any recommendations for shows?
I recently watched an interesting movie at the theater.	That’s great! What type of movie was it?

### Sustainability and Energy Use

We quantified the on-device energy use by sampling the GPU board power at 1 Hz using the NVIDIA Management Library and integrating the trace, excluding the one-time model load. For the LENOHA ST classifier (nongenerative FAQ pathway), the energy consumption was 0.445 Wh per 200 inputs, corresponding to approximately 2.23 mWh per request (latency≈0.10 s; video RAM≈2.2 GiB average, ≈2.5 GiB peak). In contrast, the local SLM pathway (Swallow-8B) consumed 33.65 Wh per 200 replies, corresponding to approximately 168 mWh per reply (latency≈8.51 s; video RAM≈13.3 GiB average, ≈14.0 GiB peak).

Thus, the nongenerative clinical pathway is approximately 75 times more energy efficient than the generative small-talk pathway, despite operating on the same hardware (Table 4). We also report, for context, a comprehensive cloud figure for a single text prompt, which is approximately 240 mWh (including idle power, CPU, RAM, and power usage efficiency) [29]. Because the measurement methods differ, these values should not be interpreted as a strict like-for-like comparison; however, they provide objective context indicating that a locally routed, nongenerative path is substantially more energy-efficient.

**Table 4.** Energy and latency comparison of local nongenerative, local generative, and cloud-generative settings.

System	Parameters (billion)	Model	Environment	Measurement method	Energy (mWh/request)	Latency (s)
LENOHA^a^ ST^b^ classifier (nongenerative, FAQ)^c^	0.56	Nongenerative	Local	Device-integral (GPU^d^ power integration)	2.23	0.1008
LENOHA SLM^e^ generation (small-talk, 8B)	8	Generative	Local	Device-integral (GPU power integration)	168	8.5147
Google Cloud: median Gemini text prompt [26]^f^	N/A^g^	Generative	Cloud	Comprehensive (idle/CPU/RAM/data-center PUE^h^ included)	240	N/A

a LENOHA: Low Energy, No Hallucination, Leave No One Behind Architecture.

b ST: sentence transformer.

c FAQ: frequently asked question.

d GPU: graphics processing unit.

e SLM: small language model.

f The cloud value reflects 0.24 Wh (240 mWh) per text prompt (comprehensive, including idle/CPU/RAM/data-center PUE≈1.09) [29]. Because the measurement methods differ between local device-integral and cloud-comprehensive reports, these values provide a contextual reference and should not be overinterpreted as a strict like-for-like comparison.

g N/A: not applicable.

h PUE: power usage effectiveness.

### Failure Analysis

Table 5 lists examples of the E5-large-instruct misclassifications. Qualitative analysis of these errors revealed 2 distinct patterns. First, false positives occurred when casual scheduling or holiday inquiries (eg, “Are you available at the end of this month?”) were misclassified as clinical questions. This pattern results in sending nonclinical utterances to the deterministic FAQ pathway, which, while reducing conversational naturalness, remains acceptable under the safety-first architecture.

**Table 5.** Examples of misclassified utterances by E5-large-instruct.

Patient utterance (English translation)	Ground truth	E5-instruct prediction
Are there any medical conditions for which I should be cautious?	Clinical question	Casual
Should I remove my dentures?	Clinical question	Casual
What should I do if I experience numbness in my tongue or lips?	Clinical question	Casual
Do you have any plans for the upcoming holidays?	Casual	Clinical question
Are you available at the end of this month?	Casual	Clinical question
Did you take time off during the New Year’s holidays?	Casual	Clinical question

Conversely, false negatives occurred when specific clinical queries (eg, inquiries about postoperative numbness, dentures, or underlying medical conditions) were misclassified as casual conversation. Routing these medical inquiries to the generative small-talk pathway bypasses the intended structural safeguards of the system. Although the overall error rate was extremely low, these specific misclassifications highlighted critical edge cases in which semantic similarities blurred the decision boundary. This indicates that further optimization of the cosine similarity threshold or the implementation of a secondary keyword-based safety net is necessary to ensure zero leakage of safety-critical questions in future deployments.

## Discussion

### Principal Findings

The principal finding of this study is that a locally executable ST classifier can achieve near-perfect accuracy in separating clinical queries from casual conversations, matching the performance of frontier cloud LLMs while using approximately 1/75th of the energy.

By evaluating this safety-first dialog architecture (LENOHA) across 2 clinically distinct preprocedural settings—oral and maxillofacial surgery, and upper gastrointestinal endoscopy—we showed that lightweight, locally executable models can achieve exceptional discrimination performance. This finding is important because it demonstrates that high-precision filtering of patient utterances does not necessarily require large, energy-intensive models or external data transmission.

### Architectural and Sustainability Implications

The second contribution is architectural. Many current health care chatbot designs attempt to “do everything” using a single generative model. However, as demonstrated by Safrai and Azaria [30], mixing casual dialog with clinical queries in a single prompt can lead to “context contamination,” severely degrading the model’s medical reasoning. By contrast, LENOHA enforces a hard separation: if the input is clinical, the system does not generate a response; instead, it returns a canonical, clinician-authorized FAQ answer. Only low-risk, nonclinical inputs are routed to the SLM.

This path is conceptually simple but highly aligned with implementation checklists, such as those proposed by Morley et al [19], and recently established digital health ethics frameworks, such as SAFE-AI [26], because it keeps epistemic uncertainty low, preserves institutional control over the content, and makes auditability straightforward. To address the risks of context contamination, there is a growing recognition that structural safeguards are required. A highly relevant emerging standard is the MCP, which mitigates such vulnerabilities by architecturally compartmentalizing the input data [15]. Unlike standard open-ended prompting, MCP strictly formats and isolates the context provided to the model, reducing the likelihood that extraneous details or casual “small talk” will be misinterpreted as clinical facts [16]. Our dual-pathway design shares this fundamental architectural philosophy. By enforcing a strict separation between clinical retrieval and casual generation, our approach illustrates how rigid architectural constraints—rather than relying solely on model training or prompt engineering—can play a central role in maintaining clinical safety and preventing reasoning degradation.

Energy and sustainability considerations further support this design. Our measurements showed that the nongenerative, FAQ-returning path consumed approximately 2.23 mWh per request, whereas local small-talk generation with an 8B model cost approximately 168 mWh per request. By routing safety-critical clinical queries to a deterministic, low-energy module and reserving high-energy generative inference only for low-risk, nonclinical small talk, our hybrid architecture substantially reduces operational carbon debt. Based on the FY2023 Japanese national average emission factor (0.423 kg-CO₂/kWh), this corresponds to roughly 0.94 mg-CO₂ vs 71.1 mg-CO₂ per interaction—a 75-fold reduction in the per-request carbon footprint [31]. This design directly addresses the calls for more sustainable natural language processing practices and helps ensure that the digital transformation of health care does not come at the cost of environmental integrity.

In other words, most of the energy cost lies in the “nice-to-have” conversational layer, not in the safety-critical layer. An architecture that allows clinics to serve 100% of clinical queries on the low-energy path and reserves the high-energy path only for casual rapport-building is, therefore, substantially more scalable for facilities with limited GPUs, limited power budgets, or intermittent connectivity. This is particularly relevant for regions with many inhabited remote islands and persistent difficulties in accessing specialists; in such settings, a locally executable, bandwidth-independent system is not only privacy-preserving but also feasible.

Our research base in Nagasaki Prefecture includes 51 inhabited islands with approximately 110,000 residents, representing one of Japan’s highest concentrations of remote islands. Such regions exemplify the urgent need to bridge health care access gaps, as many residents face substantial difficulties in accessing specialized medical services. In underserved island regions where the specialist workforce and reliable connectivity are limited, such an architecture could support clinicians by offloading routine preprocedural communication while preserving privacy and feasibility, thereby helping to reduce the working time burden on medical specialists, especially because the system can operate continuously throughout the day.

A recent study by Wewetzer et al [32] highlighted that the implementation of AI-supported health care in rural areas faces significant barriers, primarily due to technological limitations and a pronounced lack of patient trust in AI compared to urban populations.

### Limitations

Several limitations should be considered when interpreting the findings of this study.

First, this study deliberately adopted a text-based interface and did not integrate ASR. Our preliminary tests indicated that the current ASR error rates for Japanese clinical dialog could obscure the performance of the core classification architecture; therefore, a rigorous ASR-inclusive evaluation is deferred to future work. This choice improves internal validity but limits immediate use in fully speech-based encounters, particularly in dialectal or low-audio-quality settings. Fairness and safety considerations in ASR for health care were major factors in this decision.

Second, we focused on the performance of the input-filtering (classification) module rather than an end-to-end user experience. This reflects a safety-first philosophy: before assessing rapport, empathy, or patient satisfaction, it is ethically necessary to demonstrate that the system can reliably separate high-risk clinical questions from low-risk casual conversations. Evaluating whether the chatbot can support complex social or emotional roles was beyond the scope of this foundational work and will require qualitative, context-sensitive studies. Our classifier relies on externally developed models for sentence embeddings and thus inevitably inherits any representational biases encoded in the model’s pretraining data. In our architecture, however, these biases can only affect the routing decision (ie, whether an utterance is classified as clinical or casual) and cannot alter the content of clinical advice itself, which is strictly constrained to clinician-authored FAQ texts. We partly mitigated setting-specific overfitting by validating and testing the classifier across 2 distinct preprocedural domains; however, we did not perform a formal audit of E5-large’s subgroup biases in Japanese patient speech, which remains an important area for future work.

Third, this study used standard Japanese to establish a robust performance baseline. Our pilot experiments indicated that optimizing the cosine similarity thresholds for the ST requires precise tuning, and introducing linguistic noise (eg, dialects or slang) at this initial stage could obscure the true performance of the routing architecture. Having now demonstrated that the system achieves high separation accuracy (AUC >0.99) under controlled conditions, future work will focus on expanding the system’s robustness to diverse patient personas, including those with low health literacy or regional dialects.

From a life cycle perspective, our study is best understood as an early-phase study. In line with step-wise implementation models, such as those of van de Sande et al [27], this study primarily covers phase 1 (AI model development) and phase 2 (assessment of AI performance and reliability), rather than phase 3 (clinical testing of AI with actual patients). This is also consistent with broader AI life cycle concepts proposed by Kuziemsky et al [28], which emphasize that development and validation phases should precede in-workflow clinical evaluation. Furthermore, this approach intentionally aligns with the latest core requirements for ethical AI implementation proposed by Morley et al [19], which mandate the establishment of “epistemic certainty” and “validated outcomes” before real-world deployment. Accordingly, we used expert-supervised synthetic utterances to isolate routing performance and energy use under controlled conditions, and we do not yet claim full clinical readiness; a phase-3, patient-facing evaluation in real clinical workflows remains an important next step.

### Conclusions

The main insight from this study is that the performance of clinical AI is not solely a function of computational scale. Human clinical expertise—practice-based experiential knowledge that is often underrepresented in web-derived training corpora—remains central to safety and reliability. Consistent with recent studies on clinical prediction, our findings illustrate that comparable task performance can be obtained without relying on frontier LLMs when such expertise is carefully encoded and constrained, highlighting a knowledge-centric, complementary path alongside scale-driven model development [33,34].

Taken together, these findings support a dual-pathway design for medical dialog systems: (1) a generation-free clinical pathway that limits medical queries to auditable retrieval (eg, verbatim FAQ answers) and rejects or defers unverifiable inputs, and (2) a dedicated small-talk pathway that delivers brief, structured, empathic interactions through a lightweight generative model.

## Supplementary material

10.2196/89173 Multimedia Appendix 1 Clinical schema and annotation guidelines.

10.2196/89173 Multimedia Appendix 2 Prompts used for synthetic data generation.

10.2196/89173 Multimedia Appendix 3 Complete test performance metrics, including 95% CIs.

## Abbreviations

ASR: automatic speech recognition
AUC: area under the curve
FAQ: frequently asked question
GPU: graphics processing unit
LENOHA: Low Energy, No Hallucination, Leave No One Behind Architecture
LLM: large language model
MCP: Model Context Protocol
RAG: retrieval-augmented generation
SAFE-AI: Scalable Agile Framework for Execution in AI
SBERT: sonoisa/sentence-bert-base-ja-mean-tokens
SLM: small language model
ST: sentence transformer
