# Supplementary material for: A Locally Executable AI System for Improving Preoperative Patient Communication: Multidomain Clinical Evaluation
Source: JMIR Med Inform. 2026 Jul 21;14:e89173. doi: 10.2196/89173 (PMC13386661; doi:10.2196/89173)
Supplement: Multimedia Appendix 1 [file medinform-v14-e89173-s001.docx]

**Supplementary File 1: Clinical Schema and Annotation Guidelines**

**Overview**

This document outlines the operational definitions used by human experts to label the datasets (Ground Truth) and presents the structural schema of the clinician-curated FAQ database. This strict separation ensures that LLM hallucination risks are structurally mitigated.

**1. Annotation Definitions (Classification Criteria)**

To rigorously separate utterances that require medical oversight from those that do not, the following definitions were applied across all domains.

**[Clinical Questions]**

Utterances explicitly seeking medical, clinical, or practical judgments and information.

- **Examples of intent:** * Questions about post-procedural life, work, or diet.
  - Medical concerns regarding anesthesia, bleeding, pain, or infection.
  - Specific logistical queries (duration, cost, precautions).
  - Consultations regarding regular medications, comorbidities, or pregnancy/breastfeeding.
  - Questions about procedure cancellation, timing, or pre-procedural preparation.
  - Consultations regarding emergency contacts, *Helicobacter pylori*, or follow-up plans.
  - Requests for specific medical advice or instructions from healthcare staff.

**[Casual Conversations (Small Talk)]**

Utterances whose primary intent is NOT to seek direct medical or clinical information.

- **Examples of intent:**
  - Greetings, weather discussions, or general small talk.
  - Remarks about the visit (e.g., transportation, waiting time).
  - Personal updates (excluding medical symptom consultations).
  - **Simple expressions of emotion** (e.g., "I'm nervous," "I'm scared," "I'm anxious") that do not explicitly ask for medical coping strategies.
  - Light expressions of gratitude.
  - Expressions of enthusiasm or readiness for the procedure.
- **Key Decision Boundary:** The critical criterion is whether the speaker expects a clinically aligned medical response. For instance, "I am nervous" is classified as Casual (Small Talk), whereas "Are there any ways to ease my nerves?" is classified as a Clinical Question requiring a safe, vetted response.

**2. Clinical FAQ Schema Template**

The following structural framework was used by domain experts (dentists/oral surgeons and gastroenterologists) to categorize the vetted FAQ database. Utterances classified as "Clinical Questions" above are deterministically routed to one of these verified categories.

| **Major Category Group** | **Sub-category Examples (Used across domains)** |
| --- | --- |
| **Procedural Details** | Anesthesia, surgery duration, specific steps, sedation protocols. |
| **Post-operative Care** | Bleeding, swelling, pain management, dietary restrictions, medication. |
| **Logistics & Admin** | Cost, scheduling, cancellation, insurance coverage, hospital rules. |
| **Daily Life Impacts** | Return to work, physical exercise, travel, pregnancy/breastfeeding. |
| **Complications/Risks** | Infection, numbness (dysesthesia), TMJ issues, emergency contacts. |
| **Domain-Specific** | *H. pylori* queries (Gastroscopy) / Wisdom tooth specifics (Extraction). |
