# Supplementary material for: A Locally Executable AI System for Improving Preoperative Patient Communication: Multidomain Clinical Evaluation
Source: JMIR Med Inform. 2026 Jul 21;14:e89173. doi: 10.2196/89173 (PMC13386661; doi:10.2196/89173)
Supplement: Multimedia Appendix 2 [file medinform-v14-e89173-s002.docx]

**Supplementary File 2: Prompts Used for Synthetic Data Generation**

**Overview** To ensure the reproducibility of our synthetic validation and test datasets, we utilized structured prompts across multiple frontier LLMs. The generation process strictly separated "Clinical Questions" and "Casual Conversations" using two distinct prompts. The LLMs were instructed to act as patients and use standard Japanese to establish a clean baseline without dialectal noise.

Below are the exact prompt templates (translated to English for review, with original Japanese prompts provided). The examples below are for the tooth extraction domain; the gastroscopy domain used the exact same structure with adapted medical categories.

**1. Prompt for Generating "Clinical Questions"**

**English Translation of Constraints:**

- **Role:** You are a patient scheduled for oral surgery.
- **Objective:** Collect clinical questions that a patient would ask a doctor before a tooth extraction (especially wisdom teeth).
- **Condition:** Limit questions to medical/practical content that requires a "clear explanation or judgment by a doctor." Use grammatically correct, standard Japanese (no dialects).
- **Categories to include:** Travel, postoperative life, jaw, anxiety, anesthesia, dysesthesia, general anesthesia, return to work, bleeding, diet, swelling, pain, indication, procedure time, bone resection, doubts, medication, cooling, TMJ disorder, infection, cost, prosthetics, pregnancy/breastfeeding, comorbidities, cancellation, presence of wisdom teeth, tooth longevity, suture removal.

**2. Prompt for Generating "Casual Conversations (Small Talk)"**

**English Translation of Constraints:**

- **Role:** You are a patient who just received an explanation for a tooth extraction at a dental clinic.
- **Objective:** Collect examples of common small talk or casual remarks made to doctors, medical staff, or receptionists before the extraction.
- **Condition:** Do NOT include any specialized questions from the clinical categories (list of clinical categories provided to strictly exclude them). Remarks can be completely unrelated to the extraction, such as "small talk," "trends," "politics," "economy," or "family." Use grammatically correct, standard Japanese.
